# Structured headache services as the solution to the ill-health burden of headache: 1. Rationale and description

**DOI:** 10.1186/s10194-021-01265-z

**Published:** 2021-07-21

**Authors:** Timothy J. Steiner, Rigmor Jensen, Zaza Katsarava, Lars Jacob Stovner, Derya Uluduz, Latifa Adarmouch, Mohammed Al Jumah, Ali M. Al Khathaami, Messoud Ashina, Mark Braschinsky, Susan Broner, Jon H. Eliasson, Raquel Gil-Gouveia, Juan B. Gómez-Galván, Larus S. Gudmundsson, Akbar A. Herekar, Nfwama Kawatu, Najib Kissani, Girish Baburao Kulkarni, Elena R. Lebedeva, Matilde Leonardi, Mattias Linde, Otgonbayar Luvsannorov, Youssoufa Maiga, Ivan Milanov, Dimos D. Mitsikostas, Teymur Musayev, Jes Olesen, Vera Osipova, Koen Paemeleire, Mario F. P. Peres, Guiovanna Quispe, Girish N. Rao, Ajay Risal, Elena Ruiz de la Torre, Deanna Saylor, Mansoureh Togha, Sheng-Yuan Yu, Mehila Zebenigus, Yared Zenebe Zewde, Jasna Zidverc-Trajković, Michela Tinelli, on behalf of Lifting The Burden: the Global Campaign against Headache

**Affiliations:** 1 Department of Neuromedicine and Movement Science, NTNU Norwegian University of Science and Technology, Edvard Griegs gate, Trondheim, Norway; 2 Division of Brain Sciences, Imperial College London, London, UK; 3 Danish Headache Centre, Department of Neurology, University of Copenhagen, Rigshospitalet Glostrup, Glostrup, Denmark; 4 Evangelical Hospital Unna, Unna, Germany; 5 Department of Neurology, University of Duisburg-Essen, Essen, Germany; 6 EVEX Medical Corporation, Tbilisi, Georgia; 7 IM Sechenov First Moscow State Medical University (Sechenov University), Moscow, Russian Federation; 8 Norwegian Advisory Unit on Headache, Department of Neurology and Clinical Neurophysiology, St Olavs University Hospital, Trondheim, Norway; 9 Neurology Department, Cerrahpaşa School of Medicine, Istanbul University, Istanbul, Turkey; 10 Community Medicine and Public Health Department, Cadi Ayyad University School of Medicine, Marrakech, Morocco; 11 Department of Neurosciences, King Fahad Medical City, MOH, Riyadh, Saudi Arabia; 12 King Saud Bin Abdulaziz University for Health Sciences, Riyadh, Saudi Arabia; 13 King Abdulaziz Medical City, Riyadh, Saudi Arabia; 14 Headache Clinic, Neurology Clinic, Tartu University Hospital, Tartu, Estonia; 15 Weill Cornell Medicine Headache Program, Department of Neurology, Weill Cornell Medical College, New York, NY USA; 16 Department of Neurology, Centralsjukhuset, Kristianstad, Sweden; 17 Headache Centre, Neurology Department, Hospital da Luz, Lisbon, Portugal; 18 Hospital de Sant Joan Despí Moisès Broggi, Barcelona, Spain; 19 Faculty of Pharmaceutical Sciences, School of Health Sciences, University of Iceland, Reykjavik, Iceland; 20 Department of Anesthesiology, Medical College of Georgia, Augusta University, Augusta, GA USA; 21 Department of Paediatrics, University Teaching Hospital, Lusaka, Zambia; 22 Laboratory of Clinical and Experimental Neuroscience, Faculty of Medicine, Université Cadi Ayyad Marrakech, Marrakech, Morocco; 23 Department of Neurology, University Teaching Hospital Mohammed VI, Marrakech, Morocco; 24 Department of Neurology, National Institute of Mental Health and Neurosciences (NIMHANS), Bangalore, India; 25 Department of Neurology and Neurosurgery, The Ural State Medical University, Yekaterinburg, Russia; 26 International Headache Centre “Europe-Asia”, Yekaterinburg, Russia; 27 Neurology, Public Health, Disability Unit, Fondazione IRCCS Istituto Neurologico Carlo Besta, Milan, Italy; 28 Tjörn Headache Clinic, Rönnäng, Sweden; 29 Department of Neurology, Mongolian National University of Medical Sciences, Ulaanbaatar, Mongolia; 30 Faculty of Medicine, University of Technical Sciences and Technologies, Bamako, Mali; 31 Department of Neurology, University Hospital of Neurology and Psychiatry “St Naum”, Medical University Sofia, Sofia, Bulgaria; 32 1st Neurology Department, Aeginition Hospital, Medical School, National and Kapodistrian University of Athens, Athens, Greece; 33 Chief of Department of Health Organization, Ministry of Health, Baku, Azerbaijan; 34 Moscow Research Clinical Centre for Neuropsychiatry, Moscow, Russian Federation; 35 University Headache Clinic, Moscow, Russian Federation; 36 Department of Neurology, Ghent University Hospital, Ghent, Belgium; 37 Institute of Psychiatry, University of São Paulo, Hospital Albert Einstein, São Paulo, Brazil; 38 Department of Neurology, Hospital Luis Negreiros Vega, Callao, Lima Peru; 39 Department of Epidemiology, National Institute of Mental Health and Neurosciences (NIMHANS), Bangalore, India; 40 Department of Psychiatry, Kathmandu University School of Medical Sciences (KUSMS), Dhulikhel, Kavre Nepal; 41 Dhulikhel Hospital, Kathmandu University Hospital, Dhulikhel, Kavre Nepal; 42 European Migraine and Headache Alliance, Valencia, Spain; 43 Department of Neurology, Johns Hopkins University School of Medicine, Baltimore, MD USA; 44 Department of Internal Medicine, University Teaching Hospital, Lusaka, Zambia; 45 Neurology Ward, Sina Hospital, School of Medicine, Tehran University of Medical Sciences, Tehran, Iran; 46 Headache Department, Iranian Center of Neurological Researches, Institute of Neuroscience, Tehran University of Medical Sciences, Tehran, Iran; 47 International Headache Centre, Department of Neurology, Chinese PLA General Hospital, Beijing, China; 48 Department of Neurology, School of Medicine, College of Health Sciences, Addis Ababa University, Addis Ababa, Ethiopia; 49 Neurology Clinic, Clinical Centre of Serbia, Belgrade, Serbia; 50 Care Policy and Evaluation Centre, The London School of Economics and Political Science, London, UK

**Keywords:** Headache disorders, Public health, Health policy, Barriers to care, Needs assessment, Health-technology assessment, Structured headache services, Service organization and delivery, Primary care, Global Campaign against Headache

## Abstract

In countries where headache services exist at all, their focus is usually on specialist (tertiary) care. This is clinically and economically inappropriate: most headache disorders can effectively and more efficiently (and at lower cost) be treated in educationally supported primary care. At the same time, compartmentalizing divisions between primary, secondary and tertiary care in many health-care systems create multiple inefficiencies, confronting patients attempting to navigate these levels (the “patient journey”) with perplexing obstacles.

High demand for headache care, estimated here in a needs-assessment exercise, is the biggest of the challenges to reform. It is also the principal reason why reform is necessary.

The structured headache services model presented here by experts from all world regions on behalf of the Global Campaign against Headache is the suggested health-care solution to headache. It develops and refines previous proposals, responding to the challenge of high demand by basing headache services in primary care, with two supporting arguments. First, only primary care can deliver headache services equitably to the large numbers of people needing it. Second, with educational supports, they can do so effectively to most of these people. The model calls for vertical integration between care levels (primary, secondary and tertiary), and protection of the more advanced levels for the minority of patients who need them. At the same time, it is amenable to horizontal integration with other care services. It is adaptable according to the broader national or regional health services in which headache services should be embedded.

It is, according to evidence and argument presented, an efficient and cost-effective model, but these are claims to be tested in formal economic analyses.

## Introduction

Governments, politicians and health-service managers concerned about the cost of headache care for very large numbers of people fail to recognize a fundamentally important aspect of the economics of headache disorders: untreated, they are a huge financial drain. The high levels of disability repeatedly attributed to headache, and migraine in particular [1–9], are expressed not only as lost health but also in lost productivity [10–15] and detriments to gross domestic product (GDP) [16–19].

In an enlightened view, this is an opportunity. A wealth of evidence attests the efficacy of treatments for migraine and other primary headache disorders that can well be provided by non-specialists [20]. In a reasonable expectation, good health care delivering these treatments efficiently to those who will benefit from them will substantially reduce the ill-health burden of headache. The costs may be high – because there are very many such people – but, again in a reasonable expectation, interventions achieving this purpose will be cost-effective [21]. In many economies they may be cost saving, through the recovery of lost work time [22].

Regrettably, throughout the world, the opportunity is missed: health-care systems that ought to provide this care either do not exist or, where they do, fail to reach many who need it [23, 24].

In this manuscript, a product of the Global Campaign against Headache [24–26], we aim to show the solid basis of these expectations. In so doing, we aim, more pertinently, to generate political recognition of the need to address this health-care failure and the educational failures lying behind it [22]. Further, by setting out a model of *how* they can be addressed, we lay the foundations for economic analyses demonstrating the *value* of treating headache.

## Methods

Experts from all world regions, in headache, health service organization or health-technology assessment, were brought together to contribute to these proposals through email correspondence. The group was diverse, with members drawn from high-, middle- and low-income countries.

They took evidence from the published literature and, using this, built a headache-care model by developing and refining previous proposals for headache service organisation [27–29] put forward by *Lifting The Burden* (LTB) [26] and the European Headache Federation (EHF) [30]. They extended the applicability of the model beyond Europe through their own expertise and local knowledge and by drawing from experience and understanding gained by the Global Campaign against Headache during its 16 years of activities worldwide [26].

## The problems

### The challenge of numbers

Headache disorders are common, and, although most are episodic, in many cases they are lifelong conditions. They are also acknowledged to be among the top three causes of disability in the world [5–9]. Three – migraine, tension-type headache (TTH) and medication-overuse headache (MOH) – account for almost all headache-attributed burden [1, 5–8]: burden expressed in personal suffering, disability, impaired quality of life and financial cost as well as impact extending beyond those immediately affected [10].

Given all of this, it is unsurprising that large numbers of people with headache seek health care [31–33]. For example, in a United Kingdom (UK) study based in primary care 20 years ago, 17% of registered patients aged 16–65 years had consulted a general practitioner (GP) because of headache [33]. This is a good example because, in the UK, virtually everyone is registered with a GP.

However, existence of a health disorder, even one that is manifestly burdensome, does not translate directly into *need* for professional health care. *Need* in the context of health economics and policy is defined with regard to potential for benefit. Effective treatments exist [20, 34], but the proposal that everyone with headache has need for professional care is not arguable in a resource-limited world. Need is predicated on anticipated benefit, but this must rise above a *threshold of benefit*, itself judged against benefit achievable by other means (cost-effectiveness).

#### Headache-related health care needs assessment

Thresholds are hard to set objectively, although needs assessments are highly sensitive to them.

A previous estimate – essentially based on expert opinion – is that 50% of people with headache can manage themselves, using, if necessary, simple over-the-counter (OTC) medications [28]. They do not, or rather should not, need professional health care. While this estimate reflects the proportion of successful outcomes in clinical trials of OTC medications, these have almost all been conducted in *patients*, who are not the relevant denominator.

Many people do manage themselves, a large proportion through necessity, others from choice, none of them necessarily effectively. Those who choose self-management are not only the less severely affected [35]: they include a number who, for whatever reason and despite significant disability [36, 37], expect the marginal benefit of professional involvement in their care to be small (sub-threshold benefit negates need). Here is a problem, because expectations are quite often unrealistic – too high, or sometimes too low – so that needs assessment based on *what people currently do* [31–33, 35–47] has very questionable validity. This is more so when service improvement is planned: a better service – if “better” means delivering enhanced benefit – should see greater usage than a poor service it replaces (*discovered* need). While planning must factor this in, it is difficult to estimate.

Aside from these consumer-driven issues, another is also threshold-dependent. Cash-limited health services seek *value for money*: they will discount assessed needs, however great, whenever utility gain per unit of health-care resource consumption will be low. In headache medicine, the potential for benefit from professional health care is generally greatest among those worst affected, so that health policy might reasonably focus on these. Further, both migraine and MOH can, in most cases, be treated not only effectively but also at rather low cost [20, 21].

What follows – an assessment of how much professional headache care should be provided as a matter of policy – updates earlier estimates [27–29]. The approach adopted is conservative: it will under- rather than overestimate need. As will become apparent, any other approach would be unhelpful.

As with all economic assessments, there are many assumptions.

The first is that *only those with disabling headache need professional care*. The implication that others can adequately look after themselves is possibly unfair, but the assumption respects a reasonable view of priority. It effectively and helpfully removes most people with episodic TTH from consideration, this disorder generally (again perhaps unfairly [48]) being regarded as not significantly disabling [49] (There is also little that professional care can do for most episodic TTH beyond offering OTC medications [20]).

About two thirds (66%) of the world’s population are aged 15–64 years [50], these being the years during which headache disorders tend to be troublesome. About 25% are aged 14 or under [51]. Thus, with regional variations, in every million people living in the world there are 660,000 and 250,000 in these age groups respectively. Primary headache is less common, and less troublesome, in older people [1], who therefore contribute negligibly to expected numbers.

Best current epidemiological evidence suggests that a global average of about 15% of adults aged 15–64 have migraine [1, 6]. Further evidence is that 80% of these 15% (*ie*, 12%) are significantly disabled through pain and associated symptoms [52]. In every million people in the world, therefore, there are 80,000 adults (12% of the 660,000 aged 15–64 years) who need care because of migraine-attributed disability. A small proportion of adults have chronic TTH. While this is likely to be disabling, their numbers are not reliably known, partly because epidemiological data are limited and partly because conflation with MOH makes these data unreliable. As for MOH, the proportion varies greatly from country to country, with the best and most recent estimate suggesting a global mean prevalence of 1.5% [53]: another 10,000 (1.5% of the 660,000) needing care for two reasons. MOH is rated as highly disabling [49, 54], not surprisingly since it is frequently recurring by definition and very painful when present [55]. Although medication overuse may be the means by which many people with MOH nonetheless remain (partly) functional, it always requires professional care because it will not otherwise resolve.

The total of 90,000 per million (13.5% of the 660,000), which ignores TTH for reasons stated, is only one third of the estimated prevalence of all headache including TTH [1, 6] – substantially less than the 50% proposed earlier as being the proportion in need of professional care [28]. It may be over-conservative: it is somewhat below the UK finding (above) that 17% of GP-registered adults consult for headache [33], but this, although essentially a population-based observation, was a reflection of *demand*, not *need*. These, as we argue below, are not the same.

Needs arise in the child and adolescent populations also, but are more difficult to quantify, partly because there are fewer data – and none that are reliable in very young children (below 6 years) [1, 56]. In the age range 6–14 years, headache is apparently as common as in adults [1, 56, 57], but it has different characteristics. While *migraine* prevalence is lower, dependent upon age and reaching adult levels during the course of adolescence, *undifferentiated headache* (UdH) largely fills its place, albeit with less disabling consequences [57]. Furthermore, even migraine in these age groups tends to be short-lasting. In the absence of better data, a conservative but reasonable working basis is that headache-care needs in these age groups, in terms of numbers, are, proportionately, half those of adults [28, 29]: another 17,000 (0.5*13.5% of the 250,000 aged 14 or under) in each million of the population.

#### Service provision requirement

From these statistics, with some further assumptions, we can make arguably fair estimates of service requirements.

First is an assumption about *demand.* Need and demand overlap, but are not the same – each can exist without the other. Need for professional headache care, defined as above (*ie*, with regard to potential for benefit), becomes demand only in those who seek care. On the other hand, care may also be sought in the absence of need (as defined). Complex and poorly understood factors govern health-care seeking behaviour and care utilization by people with headache [35]. On the negative side are the obstacles to care described below (see The “patient journey”, and “care pathway”), which act as deterrents. Failed self-management is a positive driver. False expectations – too high or too low - have positive or negative influences accordingly. Evidence suggesting that demand for headache care is expressed by as few as half of those who might be considered in need [33, 35, 36, 58] is unreliable, because studies reporting this were not well able to judge need for care. Further, it is uncertain whether needs assessment for the purpose of service provision should reflect needs expression rather than a more objective assessment of need (as we wrote earlier, needs assessment based on *what people currently do* has very questionable validity). But we will adopt this estimate, pragmatically, because no other exists. If demand is indeed suppressed by barriers to care, better and more accessible services, dismantling these barriers, will generate increase. We assume that take-up of improved services will still not be 100%, but 75%, representing a closure of half the currently estimated gap [21].

Second are multiple assumptions about time (Table 1). These are based on expert views of requirement [21, 28, 29] but tempered with conservatism. They consider only ambulatory care: inpatient management is ignored in view of the minimal requirement for it. Admission is sometimes good practice, because of comorbidities or for detoxication in MOH, but only in a tiny percentage of patients.

**Table 1:** Assumptions in estimating service requirements to meet headache-care demand in a population (updated and revised from [29])

Assumption	Argument
The average consultation need per adult patient is 1.25 h per 2 years.	This average is within a wide range of variation. In some countries (*eg*, Portugal) consultation times are subject to legal recommendations but, otherwise, consultation *need* varies mostly according to diagnosis and to level within the health-care system. Primary-care needs can usually be met by a first visit of 15–20 min’ duration for diagnosis and impact assessment followed by 10-min visits for monitoring and prescription of acute and preventative therapies, initially after 2–4 weeks then after each 3–6 months (totalling five or six in 2 years). At higher levels of care, first consultations are usually longer (up to 45 min), reflecting case-complexity, but follow-up visits are fewer.
The average consultation need per child or adolescent patient is greater: 2 h per 2 years.	Expert opinion cites the need for additional enquiry into family dynamics, schooling and peer relationships as issues relevant to management success.
No wastage occurs through failures by patients to attend appointments.	This assumption appears manifestly false, but wastage of this sort is difficult to predict in the context of proposals for service improvement. At present, it is commonly discounted by overbooking.
A health-care provider, if working full time on headache without other clinical responsibilities, is available for consultation for 1380 h/year.^a^	At any level, 1 day per week is assumed for non-clinical work (administration, audit and continuing professional development); each week therefore allows 4 days, each of 7.5 h, of patient-contact time. Only 46 weeks are worked per year.

^a^This assumption allows estimates based on *full-time equivalence* (see Table 2). It is immaterial that full-time commitment to headache is rare except in level 3

Notwithstanding the conservatism pervading the assumptions, Table 2 sets out very challenging estimates of service requirement. We say more about this below (see The health-care solution).

**Table 2:** Estimated service requirements to meet headache-related health-care demand in a population of 1 million (from [29])

Estimated numbers expressing demand^**a**^	Expected demand	
	Hours/year of medical consultation time	FTE health-care providers^b^ required to deliver
Adults: 67,500 (75% of 90,000)	42,200	37
Children and adolescents: 12,750 (75% of 17,000)	12,750	9

*FTE* Full-time equivalent; ^a^assuming 75% of those with need; ^b^one FTE provider does not necessarily imply one provider engaged full-time on headache; it could, for example, be two engaged half time on headache, or ten working 10% of full time

### The failing *status quo*: inefficient and inequitable services, and not only in low-income countries

#### The “patient journey”, and “care pathway”

Evidence from throughout the world is that headache care reaches a minority of those who need it [22, 35–40] (to say nothing about its quality).

The primary reason is a worldwide context of low priority accorded to headache disorders in the queue for health-care resources, evident for over 20 years [59]. A decade ago, the World Health Organization (WHO) published its *Atlas of Headache Disorders and Resources in the World 2011*, recording the worldwide ill health caused by headache [22]. Noting that this was mostly untreated, WHO called for change, in a message distributed to the health ministries of every country [22]. In the 10 years since, change has not been in evidence [9, 23], although attention among WHO’s member states is beginning now to fall on neurological diseases (including headache) [60].

We estimated above that two thirds of people with headache could manage themselves, needing no more than advice from pharmacists [36]. As we will show, keeping these people out of the health-care system is crucial to efficient and equitable implementation of care. But many people who might succeed in self-management lack the small amount of knowledge on which success depends, and receive neither educational nor practical support to help them. Instead, they join the queue for health care, thus, unnecessarily, embarking upon the “patient journey”.

The one third who do need professional care are likely to find the patient journey overcrowded and frustrating, with headache services fragmentary or difficult to access and the “care pathway” a mere misnomer – winding and beleaguered by *culs-de-sac* and poor signposting [61]. One consequence is direct presentation to emergency departments, without justifying medical need but benefiting the patient (at high cost) by bypassing the care pathway altogether [62, 63].

“Headache services”, if existing at all, are too often equated with headache clinics, usually located sporadically and in big urban centres according more to market forces than objective assessment of need. Public perception is often the driver, encouraged by policies in many countries that allow direct (and often unguided) self-referral to specialists. Iran, a country with well-developed health services, is a good example, with many people taking fruitless paths to neurosurgeons, otorhinolaryngologists, ophthalmologists or pain clinics (the poorly signposted care pathway [61]). In Russia, one in every three people receiving care for migraine have gone directly to neurologists [18]. In Estonia, also a country of the former USSR but with well-developed primary care and a referral system, the proportion sent by GPs to neurologists prior to an educational intervention was a not dissimilar 39.5% [64]. In Western Europe, also one in three people treated for headache in Spain, and one in four in Luxembourg, see specialists for this purpose [40]. In Greece, only one fifth of people with headache seek professional care, but most of these do so from private neurologists [65]. In the UK, where GPs maintain a gatekeeper role as an essential feature of the national health service, the proportion (9%) referred to secondary care [33] was in line with reasonable expectation (see below: *Division of caseload*). Neurologists, however, receiving most of these referrals, reported that up to a third of all their patients consulted for headache, more than for any other neurological condition [31].

Of course, specialist clinics are needed [66], but only by those with complex disorders requiring high-end multidisciplinary care, who are a small minority [28, 29]. If specialist clinics with their very limited capacity are at the centre rather than the apex of headache services, this purpose is likely to be thwarted by the overload of patients whose needs should be met elsewhere. Most people with headache have one of only a few very common disorders, which ought to be wholly familiar to primary-care providers and only rarely present diagnostic or management difficulties [20, 28]. But the underlying problem is that non-specialist care for headache is variable at best.

There are illustrative and revealing studies. To begin in high-income countries [67], in a population-based study in United States of America (USA) and UK, only two thirds of adults with migraine were found to be correctly diagnosed [35]. Half were consulting health-care providers (HCPs) – too many according to our earlier (conservative) estimate – but over 60% of those not consulting exhibited high migraine-related disability [35]. There was probable ascertainment bias in this study, but nonetheless it was indicative of malfunctioning care pathways in both countries. More recently, the Eurolight study in 10 European Union (EU) countries found that, among participants with frequent migraine and an unambiguous need for preventative medication (more than five headache days per month), fewer than 20% had received medical care at any level [68, 69]. Incorporating indices of adequacy of care, this study identified adequate acute treatment in barely half of these (*ie*, fewer than 10% in most of the countries) and even smaller proportions with the preventative medication for which they were clearly eligible [40]. In upper-middle-income Russia [67], again in a population-based survey, only 15% nationwide of people with headache were consulting, one third, as noted, with specialists [18, 41]. In lower-middle-income Nepal [67], also in a population-based survey, a much higher 58% of participants with headache had consulted a professional HCP in the previous year, and 8% had seen a specialist of some sort [42]. While these findings suggest better availability of health care in Nepal than in many other, much wealthier countries (Japan [38] and Taiwan [43], EU countries [40, 44] and UK and North America [35, 45, 46, 52]), all is not as it seems. The count of “medical consultations” in Nepal included a very wide range of HCPs, some with no counterparts or who would not be accredited as health professionals in other countries [42]. With pharmacist consultations (15%) excluded, the consulting proportion fell to 43% [42], similar to the 47% in China [47]. In the most salient comparison, with only physician-consultations considered, the proportion fell further to 19% (GPs 11%, specialists 8% [42]), much lower than those elsewhere [35, 38, 43, 45–47, 52] – except for Russia [18, 41]. Further, since there are no headache specialists and few neurologists in Nepal, “specialist” consultations were mostly with ophthalmologists, otorhinolaryngologists or psychiatrists [42]. In other words, these findings reflected high demand without indicating good care: on the contrary, headache-attributed burden in Nepal remains egregiously high [70].

### Educational failures – the root of the problem

The origins of these health-care failures are clearly traceable to educational failures, occurring at every level [22].

On the political level, few governments appear willing to take concerted action against headache [9, 23, 71]. This is puzzling [8, 22]. It indicates a lack of awareness either of its substantial population ill-health burden (increasingly unlikely, since the Global Burden of Disease [GBD] study repeatedly affirms this [2–8]) or of its equally substantial but potentially reversible economic burdens on society [11–19, 21].

Among HCPs, very limited training in headache [22] does little to engender interest, good outcomes or, ultimately, satisfaction among themselves or their patients. For doctors this is a problem sewn in medical schools, the consequence, again, of low priority: worldwide, only 4 h are committed to headache disorders in 4–6 years of formal undergraduate medical training [22].

Among the general public, headaches – neither fatal nor contagious, and often invisible – are trivialized as “normal”, and, far worse, seen by some, in those who complain of them, as no more than an excuse to avoid responsibility [23, 72].

All of these need to change.

## The health-care solution

The numbers estimated above (see Service provision requirement) cannot be regarded as precise: they are sensitive to the multiple assumptions, particularly those related to time (Table 1). But, from them, two conclusions are certain.

First of these is that specialist care, with its very limited capacity, *cannot* meet all needs, or even a substantial part of them. Systems or practice that lead to large numbers of headache referrals to neurologists or other specialists must be questioned.

Second, and the corollary to this, is that headache care in the main belongs and must be delivered in primary care. This is not undesirable for two principal reasons (others have to do with efficiency and cost containment). On a neutral level there is, as noted, no *clinical* objection to it: education may be required, but the necessary *skills* exist in primary care [20, 28]. On the plus side, since WHO’s Declaration of Alma-Ata of over 40 years ago [73], wherever health-care reform is in progress there is emphasis on strengthening primary care, with its benefits of continuity and care in the community [60, 73–77]. While models of health care vary throughout the world, whatever the overarching system of care, primary care has a recognized and important role nearly everywhere. In highly rural regions, those of sub-Saharan Africa, for example, primary care in whatever form it takes is the *only* point of contact for most patients.

### Structured headache services

#### Systems approach to health care

Drawing from engineering theory, a systems approach to a problem or need first identifies it (“understanding the problem space” – which the foregoing has done) and then pulls together system elements that, *working together*, will resolve the problem or meet the need (“the solution space”) [78]. There are steps in the process, working from needs to requirements and from design to integration to delivery [78], but these do not need to be detailed here. The key feature shared by all well-functioning systems – in health care as much as in engineering – is that “*their elements together produce results not obtainable by the same elements alone*” [78]. Applicable particularly to health care, a systems approach involves “integrating the necessary disciplines into a team who then use a structured process to deliver a system” [78]. The health-care solution to headache is envisioned through applying this approach to headache care.

Nationwide structured and educationally supported headache services embedded and integrated within a country’s health-care system are the means of efficiently, effectively and equitably mitigating the personal and societal burdens of headache to the greatest extent possible with resources available, a proposition put forward by LTB and endorsed by WHO a decade ago [22]. Structured headache services pull elements together from primary, secondary and specialist (tertiary) care, and, importantly, from pharmacy services. Equally importantly, they also harness self-management. Educational supports are an additional element, required at all system levels and including public education on when and how to make correct use of these services. Integration within the broader health-care system is crucial: if headache services merely develop ad hoc, driven not by coherent policy but opportunistically by interested individuals, as is now the case in most countries providing any service at all, they can be neither efficient nor equitable.

**Fig. 1:**
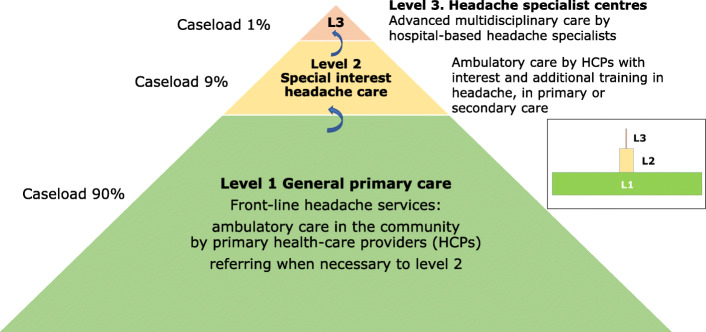
Graphic depiction of headache services organized on three levels, but based in primary care, with predicted caseloads (see text and Table 3 for explanation). Inset: arguably a better depiction

The essential purposes of headache-service organization are two-fold: to create capacity matched to need, and to divide service provision *rationally* between provider levels. The guiding principle in both purposes is that management of patients should be at the lowest level commensurate with good care. Achieving this makes most efficient use of allocated resources. Basing headache services in primary care (Fig. 1) extends reach and affords ease of access [60, 73–77]. Higher levels are reserved for those with high-frequency, complex or rare headache disorders, perhaps complicated by comorbidities that require multidisciplinary care [28, 29, 66].

#### A model of headache-service organization

The focus of headache services should be on migraine and MOH (see above: Headache-related health care needs assessment [27–29]). This does not exclude other headache disorders, but TTH, the most prevalent headache, is almost always self-manageable [20, 28, 79] while other primary headaches are far less common. As for secondary headaches, their *management* is of the causative disorder, and therefore, with a few exceptions (notably MOH), outside the ambit of headache services. But provision is needed for their *recognition*, since this is the responsibility of the services to which affected patients present – most likely to be headache services when headache is the symptom.

**Fig. 2:**
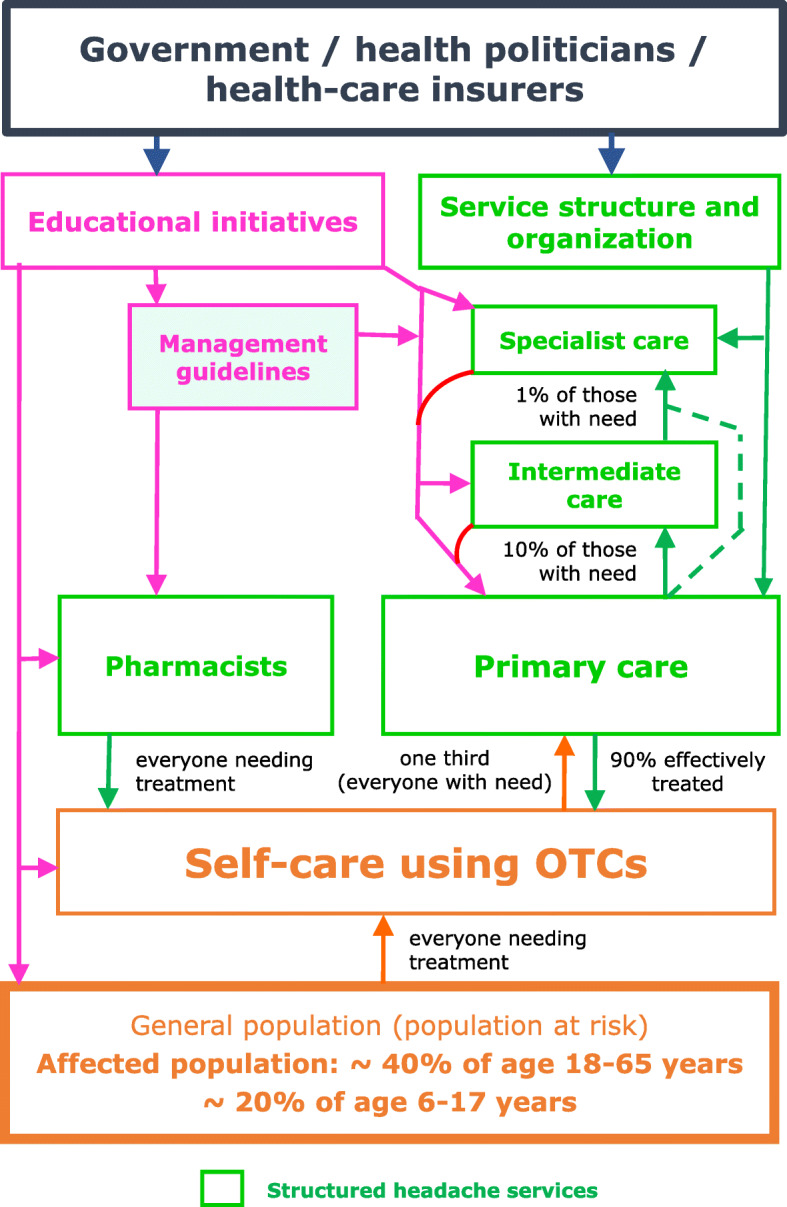
Template for structured headache services supported by educational initiatives, and expected patient flows (adapted and updated from [26, 27]; see text for explanation)

While organized headache services, if they are to be efficient and equitable, must be embedded in national or regional health services, these vary throughout the world, differently structured and not always adequately resourced. The model proposed (Fig. 2; Table 3), incorporating three interdependent levels with facilitated but controlled pathways between them while expanding the contribution from primary care, is a general guide and template adaptable to these variations. It is the interventional model adopted as the health-care solution to headache by the Global Campaign against Headache [24, 25]. The account here develops and refines earlier proposals [27–29].

**Table 3:** Headache services organized on three levels (from [29], updated from [28])

Level 1. General primary care	• front-line headache services (accessible first contact for most people with headache) • ambulatory care delivered in the community by primary health-care providers (physicians, clinical officers, nurses and/or community health workers) • referring when necessary, and (according to setting) acting as gatekeeper, to:
Level 2. Special-interest headache care	• ambulatory care delivered by physicians, clinical officers or nurses with a special interest and additional training in headache, in primary or secondary care • referring when necessary to:
Level 3. Headache specialist centres	• advanced multidisciplinary care delivered by headache specialists in hospital-based centres

##### Level 1. General primary care

Primary care is the front line of headache services (Figs. 1 and 2), available locally and accessible to all who need them. This is not everyone with headache: the estimated two thirds able to manage themselves (see above: Headache-related health care needs assessment) should do so, with advice from pharmacists and guidance from public educational programmes built into the model (see below: *Educational implications*).

Pharmacy services (perhaps level 0) need to be highlighted here. They are often greatly underutilized. Pharmacists are experts in the effects (wanted and unwanted) and uses of medications and generally the most easily accessible of all HCPs. Pharmacists can give guidance and advice not only on self-management but also on when further professional care should be sought [36].

Level 1 is provided by non-specialist HCPs, not necessarily physicians in a health-care system more dependent on other practitioners (clinical officers, nurses or community health workers, for example), although authority to prescribe is a requirement for non-OTC medications. Occupational health services can be a helpful adjunct, also provided in proximity to need (especially given the impact of headache on work). Whatever their background, HCPs providing level-1 care will need basic knowledge and understanding of headache disorders for this purpose, and many will therefore require some training in headache (see below: *Educational implications*). But the model does not require *every* HCP in primary care to offer headache services if they can share caseload between themselves according to their skills and interests, an arrangement that may be administratively easy in group practices or health centres.

Management aids designed to facilitate non-expert care are an important support to level 1 [20]. With these and the educational supports, this level should competently meet the needs of most people needing professional care for headache [28, 80]: most cases of migraine (and almost all of TTH, if needing care) can be diagnosed and managed here by HCPs who should be familiar with recommended acute and preventative drugs [30] and aware of the constraints in managing fertile women (important since these are a high proportion of people with migraine). Cluster headache, MOH and some other common secondary headache disorders listed in Table 4 should be recognized but not necessarily managed, and red-flag warnings of serious secondary headaches should also be recognized and duly acted upon [20]. Channels for referral to levels 2 and 3, urgently when necessary, should be in place for these cases, and for other patients who are diagnostically complex or difficult to manage [28, 29].

**Table 4:** ICHD-3 diagnoses [81] to be recognized at level 1 (from [29], updated from [28])

Primary headache disorders	Secondary headache disorders
1.1 Migraine without aura^a^ 1.2 Migraine with aura^a^ 1.2.3 Typical aura without headache^a^ 2.1 Infrequent episodic tension-type headache^a^ 2.2 Frequent episodic tension-type headache^a^ 2.3 Chronic tension-type headache 3.1.1 Episodic cluster headache 3.1.2 Chronic cluster headache	5.2.1 Chronic post-traumatic headache attributed to moderate or severe head injury 6.2.2 Headache attributed to subarachnoid haemorrhage 6.4.1 Headache attributed to giant cell arteritis 7.2 Headache attributed to low cerebrospinal fluid (CSF) pressure 7.4.1 Headache attributed to increased intracranial pressure or hydrocephalus caused by neoplasm 8.2 Medication-overuse headache^a^ 9.1 Headache attributed to intracranial infection 10.3 Headache attributed to arterial hypertension 11.3.1 Headache attributed to acute glaucoma 13.1.1 Classical trigeminal neuralgia

^a^Management of most of these should be within the competence of level 1

Level 1 therefore controls flow to higher levels. There is more to be said about this (see below: *The gatekeeper role within the model*).

Finally, this level should continue the long-term care of patients discharged with treatment plans (as they should be) from levels 2 or 3 [28, 29, 66, 82].

##### Level 2. Special-interest headache care

Level 2 may be in primary care, provided by HCPs (usually but not essentially GPs), but in many countries it is more likely to be offered in secondary-care polyclinics or district hospitals and by neurologists (sometimes general physicians, specialists in other fields of internal medicine or psychiatrists). Either fits the model, with training in headache to a more advanced but not specialist level (see below: *Educational implications*).

Level 2 provides more skilled ambulatory care, and has capacity only for the relatively small proportion of patients needing this (Figs. 1 and 2, and see below: *Division of caseload*). Competence should embrace the diagnosis and management of more difficult cases of primary headache, with experience in using the full range of medications. It should extend to some secondary headache disorders, though not those that are very rare [66]. Ideally, HCPs at level 2 should have access to other services such as neuroimaging, psychology and physiotherapy. Where this is not possible, and anyway for the minority of their patients outside their competence (Table 5), they require a referral channel to level 3 [28, 29].

**Table 5:** Patients likely to be referred to level 3 within optimally structured headache services^a^ (adapted from [29])

Patients with:	
• Refractory disabling headache of any type;	
• Cluster headache and other trigeminal autonomic cephalalgias, at first presentation;	
• High and low cerebrospinal fluid-pressure headaches;	
• Trigeminal and other cranial neuralgias or painful lesions of the cranial nerves;	
• Rare primary or secondary headaches;	
• Medication-overuse headache involving drugs of dependence, where personality mitigates against withdrawal of medication or where withdrawal attempts have failed;	
• Other probable or certain serious secondary headache;	
• Headaches with severe physical and/or psychological comorbidities.	
Cases of persisting diagnostic uncertainty.	
Patients in whom risk of serious underlying disorders demands specialist investigation.	
Patients who may participate in specific level-3 research projects (including clinical trials) [66].	

^a^depending in some cases on the facilities and skills available at level 2

To maintain efficiency, level 2 should repatriate patients to level 1, with management plans, as soon as is clinically appropriate.

##### Level 3. Headache specialist centres

Specialized headache centres sit at the apex of structured headache services (Fig. 1) [66].

In many countries, they remain an unfulfilled aspiration; in others, they exist – disadvantageously to them – without the lower levels in place, or are inequitably distributed. As an example of the last, a survey in Brazil identified 243 “headache specialists” in 2004/2005. These were more than twice the number estimated to be needed for Brazil’s 198 million people [83] in a well-functioning 3-level system (see below: *Division of caseload*), but 68% were in the south-east region catering for only 42.6% of the country’s population; by contrast, 12.4% were in the north-east for 28.1%, and five of Brazil’s 27 States had none [84].

The first and foremost role of specialized headache centres, as tertiary referral centres, is to manage the very small proportions of patients with primary headache disorders that are especially difficult to diagnose or treat, or with secondary headaches requiring multidisciplinary management [81], and those who for other reasons, such as comorbidities, need specialist intervention [28, 66, 82, 85, 86] (Figs. 1 and 2; Table 5). For this role, they employ accredited headache specialists or neurologists, and concentrate experience in the rare headache disorders and cranial neuralgias [28, 66]. They have access to equipment and specialists in other disciplines for the diagnosis and management of the underlying causes of all secondary headache disorders [66]. They provide limited but full-time inpatient facilities (see below: *Division of caseload*). Specialized headache centres are therefore situated within or closely affiliated (with access rights) to a university or other major hospital [66].

In their second role, level-3 centres support non-specialists at levels 1 and 2 through clinical advice, training and development of national management guidelines [66]. Sustained through bidirectional links, this is a symbiotic relationship: well-functioning lower levels reduce demand on the very limited capacity at level 3. In this role, level 3 both maintains standards throughout the system and protects its ability to perform its first role.

Repatriation of patients to level 1 (or 2) as soon as is clinically appropriate also protects this ability. Again, this should be with management plans.

Less essential additional roles, including locally mandated or broader-based research, have been described [66].

##### Provision for children and adolescents

Although their needs may differ, the model of care is the same for these age groups, with front-line services (level 1) in primary care. To the extent that neurological services provide higher levels of care, these may be replaced by child neurology or paediatric services. The latter, in some countries, are better developed than neurological services.

##### Division of caseload

Quantitative estimates of the proportions of patients with care needs at each level are largely based on expert opinion [27–29], but with some empirical support [33]. They shape the model as a broad-based pyramid with a narrow apex (Fig. 1). *Assuming all levels are in place and functioning efficiently*, they predict 90% managed at level 1 and about 10% at higher levels, with no more than 1% of all headache patients at level 3 [28] (Fig. 2).

On these estimates and the earlier assumptions about consultation times (Table 1), one full-time equivalent (FTE) HCP at levels 1, 2 or 3 can provide care at those levels to populations no larger than 25,000, 200,000 and 2 million respectively [28, 29]. (One FTE HCP does not necessarily imply one engaged full-time in headache services; it could, for example, be two HCPs engaged half time in headache, or ten engaged for 10% of full time.)

For inpatients – the very few with difficult comorbidities, or with MOH requiring detoxication – a recommended minimum is two beds per million population [28].

##### The gatekeeper role within the model

The model expands the role of primary care, and shifts demand in structured services downwards towards it – a move that is both clinically appropriate and economically efficient [60, 73–77]. This brings into focus the *gatekeeper* role of primary care as an organizational element of the model [74, 82, 87–89]. It is a controversial role.

On the one hand, patients cannot be blamed for seeking direct access to perceived experts, and gatekeeping is not a norm in many countries’ health systems. On the other, ostensibly, gatekeeping guides patients along the “care pathway”, efficiently and in their best interests through the health system according to their needs, not their demands [87]. This is a well-intentioned purpose, although arguably somewhat paternalistic. In truth, gatekeeping is the means of preventing overload in specialist services, which would deny access to some who really need it. This is a more defensible consideration. But gatekeeping is also a means of cost containment, because specialist care is costly (especially when unnecessary). Gatekeeping therefore attracts a hostile perception as a means of rationing [87–89].

How essential is it? Health systems in which gatekeeping is the norm will accommodate it easily within headache services; others may not. More important in all cases, both to effectiveness of the model [88] and to the equity of it, is efficiency at the interfaces between levels (“seams in service continuity” [82, 89]), so that no delays are system-created against those who *do* need specialist care. Efficient interfaces are matters of implementation, best determined in the context of local health services. If the model is implemented well, with all levels in place and adequate provision at each, *demand within the levels is likely to be self-regulating*, effectively governed by waiting lists.

##### Flexibility of the model

Two principal factors determine how this model might be implemented in a country (or region or district): the resources allocated to headache services and the structure of the health service accommodating them. The model has considerable flexibility, allowing adaptations in many ways without altering its intrinsic structure (Table 6).

**Fig. 3:**
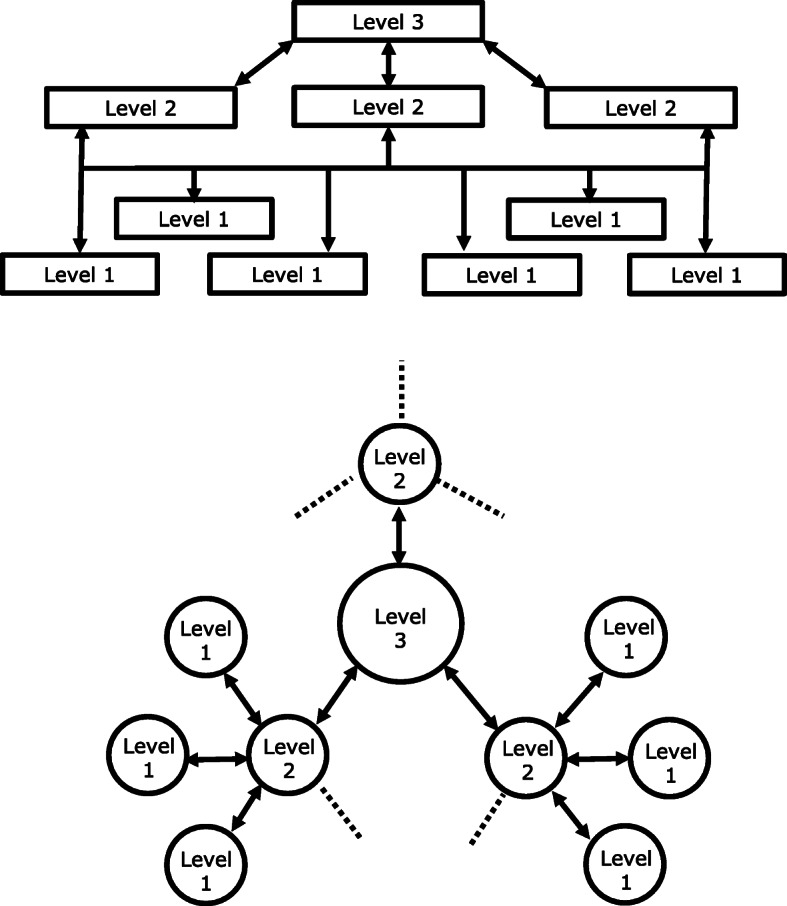
Layered (above) and hub-and-spoke (below) structures. The bi-directional arrows indicate patient flows from lower levels to higher, and clinical and educational supports from higher to lower. Each structure is amenable to top-down or bottom-up organization for integration within existing health services, although bottom-up is more likely in layered structures and top-down in hub-and-spoke. Hybrid structures are possible

**Table 6:** Adaptability of the model according to local requirements and resources (adapted from [29])

Requirement	Adaptation
Doctors vs other health-care providers (HCPs)	Many countries, as policy, are expanding the health-care roles of HCPs other than doctors. Systems in some countries may depend on service provision at level 1, and perhaps level 2, by clinical officers, nurses and/or community health workers. This is the way forward, supported by training, if the alternative is nothing.
Primary vs secondary care	Level 1 is in primary care. Level 2, on the other hand, can be in primary or secondary care: common options include neurologists or physicians (trained but non-specialist) in community or district hospitals or polyclinics.
2-level systems	Level-3 centres are in secondary care (or tertiary care in countries that make this distinction). Level 3 is therefore costly and may be unaffordable. When it cannot be fully implemented within this model, or at all, this does not detract from the benefits that can be provided to the great majority by levels 1 and 2.
Combined levels	Level 1 is by its nature community based. It is possible nonetheless, and may be appropriate, for certain level-2 centres also to offer local level-1 care. Similarly, there is no intrinsic reason why one centre cannot provide both level-2 and level-3 care.
Division of caseload	The 90:9:1% split between levels 1, 2 and 3 are estimates of need based largely on expert opinion. Throughout the world, there are variations in prevalence and characteristics of the common headache disorders, particularly in the frequency of medication-overuse headache [53]. The division of caseload between levels and capacity at each level may need adjustment, ideally based on locally gathered empirical data. The model will accommodate this without fundamental change.
Integration within existing services	The model adapts equally comfortably to layered and to hub-and-spoke structures, or hybrids of these, according to a country’s broader health-service structure. It permits bottom-up organization (patient flows driven upwards by demand at lower levels) or top-down (flows induced upwards by available capacity at higher levels) (Fig. 3).

Table 7 provides illustrations of how, with these adaptations, it might be implemented in various countries.

**Table 7:** Illustrations of how the model might be implemented, with adaptations, in various countries

Country	World Bank income level [67]	Model levels	Structure	Proposed organization (placement of levels and provision of care)	Comments
Abu Dhabi	High	3	Layered, bottom-up	Level-1 services provided by GPs in each State-owned primary health-care centre. Level-2 services provided either by GPs in selected primary health-care centres, or by hospital-based neurologists. A single level-3 centre in a specialist neurology department within a hospital-based multidisciplinary health-care facility.	Total population is about 1.5 million. There are 27 State-owned primary health-care centres, but 500 GPs, many in the private sector.
Azerbaijan	Upper-middle	3	Hybrid system, bottom-up	Level-1 services provided in remote rural areas by GPs in primary care, in urban areas by GPs in ambulatory-polyclinic services. Level-2 services provided either in the same polyclinics, where so-called district therapists/GPs can redirect to a neurologist (or to level 3), or by neurologists working in private clinics or outpatient clinics in private hospitals. Level-3 services provided in central regional hospitals with neurological beds.	Neurologists at level 2 can provide clinical and educational support to GPs in their locality in a hub-and-spoke arrangement.
Brazil	Upper-middle	3	Layered, bottom-up	Level-1 services provided in the community by GPs in primary care. Level-2 services provided by neurologists working either in the community or in hospital-based secondary care. Level-3 services provided by neurological centres, often university-based, in the larger cities.	Total population is 210 million (70% covered by public health services, 25% by supplementary health services, 5% uncovered), served by 35,000 GPs, 5000 neurologists and 500 (uncertified) “headache specialists”. Despite apparently adequate capacity at all levels (albeit unevenly distributed geographically [84]), fewer than 5% of candidates for migraine preventative drugs currently receive them [90].
Bulgaria	Upper-middle	3	Layered, bottom-up	Level-1 services provided in the community by GPs in primary care. Level-2 services provided by neurologists working either in the community or in hospital-based secondary care. Level-3 services provided by neurological centres, often university-based, in the larger cities.	Well-developed primary care operating a gatekeeper role, but GPs currently cannot prescribe many drugs without a specialist diagnosis.
China	Upper-middle	3	Hub and spoke, top down	Level-3 services provided by neurologists in provincial or university hospitals. Level-2 services provided by neurologists in county, district or municipal hospitals. Level-1 services provided by GPs in community health centres or rural clinics or hospitals.	This system is implemented in parts of the country [91]. Medical facilities at all levels provide either Western or Traditional Chinese medicine.
Colombia	Upper-middle	3	Layered, bottom-up	Level-1 services provided in the community by GPs in primary care. Level-2 services provided by neurologists in hospital-based secondary care in intermediate and larger cities. Level-3 services provided by neurological centres, often university-based, in the larger cities	Services are overseen by the State but insurance-based, provided by multiple private or public companies, each with different organizations. Currently, there is a lack of neurologists, with most located only in larger cities [92, 93]. Neurological conditions in general are not a listed priority for the health-care system [94].
Estonia	High	3	Layered, bottom-up	Level-1 services provided in the community by GPs in primary care. Level-2 services provided by neurologists working in regional/county hospitals in private or public sectors. Level-3 services provided by a subspecialty division of a university-based hospital neurology department.	Total population is about 1.3 million. There is only one university-based hospital in the country, which provides all level-3 services.
Ethiopia	Low	2	Layered, bottom-up	Level-1 services provided in the community by community health workers, nurses, clinical officers and GPs in rural health posts, local health centres and primary hospitals. Level-2 services provided by GPs, internists and neurologists working either in secondary-care general hospitals in district towns or in tertiary-care specialized university-based hospitals in larger cities.	Total population is estimated at 114 million, with the majority still using traditional medicines despite increasing health-service coverage. The gatekeeper role can be effectively integrated into the existing health-service system by training HCPs in primary care.
Georgia	Upper-middle (recently upgraded from lower-middle)	2	Layered, top down	Level-2 services provided by headache-trained neurologists in private headache clinics in major cities. Level-1 and some level-2 services provided by GPs or neurologists in urban health-care facilities elsewhere.	A system of interdependent private headache clinics currently operates outside the State system [95]: there is no State-supported alternative, and primary care is poorly developed. Level-3 centres are aspirational.
Greece	High	3	Layered, bottom-up	Level-1 services provided by GPs in private or public health-care sectors. Level-2 services provided by neurologists in private or public settings. Level-3 centres provided by headache specialists in neurology departments within hospital-based multidisciplinary health-care facilities.	Many people with headache are currently un- or under-treated. A 2018 national general population survey by the Hellenic Headache Society (HHS) found that one fifth seek professional care, most commonly from private neurologists [65]. Under the umbrella of HHS there are, currently, 14 headache centres in the public sector, three academic (level 3) [96].
Iceland	High	3	Layered, bottom-up	Level-1 services provided by GPs in primary health-care centres. Level-2 services provided by GPs with a special interest and neurologists in district health-care institutions. Level-3 services provided by headache specialists in hospitals providing specialist services.	Population is 364,000, served by 12 district health-care institutions and two university or teaching hospitals, both providing general and specialized services.
India (Karnataka State)	Lower-middle	3	Hub and spoke, top down	A single level-3 centre in the National Institute of Mental Health and Neurosciences (NIMHANS) in Bangalore. Level-2 services provided by physicians with training in headache in affiliated district or subdistrict hospitals. Level-1 services provided in urban areas by GPs in health centres and in rural areas by medical officers in community health centres and primary health centres and by health workers in subcentres. Where available, pharmacists may provide level-1 care.	State health care is primary (in community and primary health centres), secondary (in sub-district hospitals) and tertiary (in district hospitals and medical colleges). Many people favour traditional remedies. The hub-and-spoke top-down system promotes the educational and clinical supportive roles of the level-3 centre to levels below.
Iran	Upper-middle	3	Layered, bottom-up	Level-1 services provided by GPs in the community or in primary-care centres. Level-2 services provided by neurologists working either in the community or in hospital-based secondary care. Level-3 services provided by neurological centres, often university-based, in the larger cities.	Services are supported in governmental centres and hospitals, but there are also many private clinics.
Italy	High	3	Layered, bottom-up	Level-1 services provided by GPs in primary care. Level-2 services provided by hospital- or clinic-based neurologists or other specialists with interest in headache in public or private sectors. Level-3 services provided in academic hospital-based centres by headache specialists working in multidisciplinary teams.	Italy’s Health Care System is national but also regulated at its 21 regional levels. Its population of 60 million is served by more than 80 headache and migraine centres (public, private but recognized for reimbursement, or fully private). National legislation [97] recognizes chronic primary headache disorders as disabling and requiring care.
Mali	Low	3	Layered, bottom-up	Level-1 services provided by doctors, nursing assistants and health technicians in community health centres. Level-2 services provided by GPs and internists, nurses and health technicians in reference health centres at district level or in regional hospitals. Level-3 services provided by specialists in national hospitals and university hospital centres.	Mali’s health services are built on 4 levels in a pyramid structure: community health centres at level 1 (community); reference health centres at level 2 (district); regional hospitals at level 3; national and university hospitals at level 4. In Mali, health systems must accommodate simultaneous use of conventional and traditional medicines to respect long-established cultural preferences and practices. Finding the right formula to integrate these remains a challenge in health services generally.
Mongolia	Lower-middle	3	Layered, bottom-up	Level-1 services provided by GPs in primary health-care centres or soum health centres and inter-soum hospitals. Level-2 services provided by neurologists in aimag- or district-based hospitals. Level-3 services provided by neurologists in central State hospitals.	Aimags are first-level administrative divisions, soums are second-level. Total population is 3.3 million, with primary care services reaching 70%. Many people favour traditional remedies. Level 3 currently is largely aspirational because of a lack of headache specialists.
Morocco	Lower-middle	3	Hybrid system in public sector with layered bottom-up and hub-and-spoke top-down elements; top-down in private sector	Level-3 services provided by neurologists in regional and university hospitals or in private hospitals. Level-2 services provided by neurologists and GPs with special interest in district clinics or provincial hospitals or in private practices. Level-1 services provided by GPs in public primary health-care centres or private practices, or, in some rural areas, by nurses.	Morocco has a mix of HCPs: public (State-sponsored and free) and private (reimbursed through insurance or paid out-of-pocket). Primary care has a gatekeeper function, which is not always respected, while access to specialists is direct in private care. Through telemedicine, specialists in Morocco reach and advise patients living far from regional hospitals, a hub-and-spoke system that can also provide clinical and educational support to non-specialists at lower levels.
Nepal (Bagmati Province, Kavre District)	Lower-middle (recently upgraded from low)	3	Hub and spoke, top down	A single level-3 headache centre in Dhulikhel hospital, Kathmandu University Hospital (DH-KUH). Level-2 services provided by clinical officers and/or physicians in DH-KUH’s outreach health centres. Level-1 services provided by community health workers in outreach primary care centres or health posts.	Outreach health centres are around 20 in number in and around Kavre and adjoining districts. Many people favour traditional remedies. Countrywide, level 3 is currently aspirational in a geographically diverse country with major accessibility challenges [98] but, in a population survey, over half of adults with headache had engaged with HCPs of varying sorts [42], indicating capacity to build on.
Norway	High	3	Layered, bottom up	Level-1 services provided by GPs in primary care. Level-2 services provided by neurologists (and nurses) working in hospital-based neurological departments, or neurologists or GPs with special interest in headache working outside hospitals. Level-3 services provided by headache specialists and nurses in academic hospitals, working in multidisciplinary teams.	A process to establish a national system for headache care is commencing now in collaboration with the Norwegian Ministry of Health.
Pakistan	Lower-middle	3	Hub and spoke, top down	Level-3 services provided by headache-trained neurologists in private and public tertiary health centres. Level-2 services provided by neurologists in private headache clinics in larger cities. Level-1 and some level-2 services provided by neurologists and GPs in urban, suburban and rural health-care facilities and clinics.	Public health-system infrastructure is fragmented, but both private and public level-3 services exist in larger cities. Most adults seeking headache treatments go first to GPs, but direct access to specialists is available in both public and private sectors. Therefore, level-1 and level-2 services may currently be provided by neurologists or specialists. A hub-and-spoke top down model, especially with the use of telemedicine, can boost education and support for GPs and remote practice locations, thereby improving service structure and reducing inappropriate demand at level 3.
Perú	Upper-middle	3	Layered, bottom-up in public sector; unstructured in private sector	Level-1 services provided by GPs, nurses, nursing assistants and pharmacists in primary-care health centres. Level-2 services provided by neurologists in regional hospitals and private clinics. Level-3 services provided by specialist accredited neurologists (neurology services are subdivided into areas of care, including headache) in hospitals and institutes providing high-complexity care in departmental capital cities.	Perú is multicultural, with 31 million population. Its decentralized health-care system is administered by five entities (Ministry of Health, Social Health Insurance, Armed Forces, National Police and the private sector) and suffers from low investment and lack of horizontal integration. As in most of Latin America, headache has low priority. Many (doctors and general public) believe only neurologists can resolve it. Structured headache services offer solutions to these challenges, with technological supports (telemedicine can overcome geographic and economic obstacles to transfer of patients).
Portugal	High	3	Layered, bottom up	Level-1 services provided by GPs in primary health-care centres. Level-2 services provided by neurologists in referral hospitals (community hospitals). Level-3 services provided by neurologists with specialist training in headache in central and/or academic hospitals.	Well-developed occupational medicine services ally efficiently and helpfully with community health services.
Russia	Upper-middle	3	Layered, bottom up	Level-1 services provided by GPs in primary health-care centres or district-based polyclinics. Level-2 services provided by neurologists in each regional centre. Level-3 services provided by neurologists with specialist training in headache in each interregional municipal centre.	Russia has 147 million people, 565,200 physicians, 28,600 neurologists, > 50 tertiary headache centres (mostly private) and about 200 “headache specialists” [99]. Up to half of people with headache consult a physician, most often a neurologist, yet fewer than 1% with migraine use preventative medications [100]. Russia is only slowly divesting itself of arcane traditions in clinical practice, exemplified in headache management by entrenched preferences for vasoactive, nootropic and so-called neuroprotector drugs instead of evidence-based preventative drugs [101]. Ministry of Health recommendations for management of migraine [102] and mandatory inclusion of headache care in under- and postgraduate medical education are addressing care deficiencies nationally, but a headache-service implementation project in Sverdlovsk Oblast (Yekaterinburg) lacks government support [103].
Saudi Arabia (National Guard Health Affairs [NGHA])	High	2/3	Layered, bottom up	Level-1 services provided by GPs in primary health centres. Levels 2 and 3 are hospital-based, provided by neurologists, sometimes in specialized clinics.	NGHA offers one of Saudi Arabia’s health-care systems, with two medical cities, five hospitals and over 70 primary health-care centres providing full coverage for employees and their dependants. Additionally, it offers services to the general public for certain diseases and for emergencies (providing about 50% of their secondary and tertiary care, to 1.7 million people).
Serbia	Upper-middle	3	Layered, bottom up	Level-1 services provided by GPs in public health centres. Level-2 services provided by neurologists working either in a polyclinic system within the same public health centres or in local hospitals. Level-3 services provided by specialists in neurological centres, usually academic, located in the larger cities.	Well-developed primary care with a gatekeeper role but, currently, many medications cannot be prescribed by GPs without diagnosis by a neurologist, and many are not reimbursed.
Turkey	Upper-middle	3	Layered, bottom up	Level-1 services provided by GPs in public health centres. Level-2 services provided by neurologists in private or government polyclinics. Level-3 services provided by neurologists with specialist training in headache in private or government university headache centres.	Government-funded Medicaid provides free health insurance to all but is under-resourced. Not all drugs are reimbursed, while GPs in level 1 currently cannot prescribe all medications, and tend to over-prescribe analgesics. The consequence is too many patients in level-2, many with medication-overuse headache.
United Kingdom	High	3	Layered, bottom up	Level-1 services provided by GPs in each practice. Level-2 services provided by GPs with special interest (GPwSIs), sometimes with support from specialist nurse practitioners, or by neurologists based in or visiting district hospitals. Level-3 centres staffed by specialists, often supported by nurse practitioners, in neurology departments within selected multidisciplinary hospitals.	The gatekeeper role of primary care is entrenched. GPwSIs in particular fields are appointed for local areas with the purpose of avoiding unnecessary referrals to specialist care, or by local commissioning groups as local or regional leads, or by larger group practices to take a lead role within the practice [104].
United States of America (Medicaid system)	High	3	Layered, bottom up	Level-1 services provided by primary care physicians. Level-2 services provided by neurologists. Level-3 services provided by specialists within neurology departments, typically in university settings.	Medicaid (funded jointly by Federal and State governments but run by each State separately) provides free health insurance to 74 million people whose income and resources are insufficient to pay for health care. Existing Federal adult and childhood quality-of-care measures do not include headache outcome measures. Adding these could educate providers and improve usage of layered headache care, with better outcomes expected. Telemedicine referrals from rural areas could be employed between levels 1 and 2 in each State, and for follow up.
Zambia	Lower-middle	3	Layered, bottom up	Level-1 services provided in urban areas in district hospitals by clinical officers, with or without a physician, and in rural areas by clinical officers and community health workers in community health centres or rural health posts. Level-2 services provided by physicians or clinical officers in provincial or general hospitals. Level-3 services provided by neurologists in the country’s level-3 hospitals (usually university-affiliated).	These levels correspond with the 3 levels of State-provided health care, in which clinical officers substantially outnumber doctors. Level 3 is only recently a possibility with the graduation of Zambia’s first adult and paediatric neurologists. In the near-term, very small numbers of these specialists will severely limit level-3 capacity, increasing dependence on level 2.

*GP* General practitioner, *HCP* Health-care provider

##### Educational implications

*Public education* has three main purposes. Firstly, it is needed to improve people’s understanding of headache. Secondly, it should explain the use and limitations of OTC medication [79] while warning against self-mismanagement, the attendant dangers of medication overuse and the unsupervised purchase of analgesics from supermarkets and other unlicensed outlets rather than from pharmacists. Thirdly, it should explain how to make *appropriate* use of headache services, what to expect from them and, in this context, the likely futility of non-adherence [105, 106].

WHO identified *professional education* as the most pressing requirement in headache services improvement [22]. Separate educational initiatives are required at each level. Pharmacists are not generally expected to diagnose, having neither the training nor the facilities required, but they should have the knowledge to recognise treatment failures and incipient or established medication overuse [79]. Limited knowledge is required at level 1 [28, 64], but better than usually exists. Enhanced but still non-specialist knowledge is needed in level 2 [28]. In both, education should be coupled with availability – and use – of evidence-based guidelines [20, 64, 107, 108] adapted to local resource availability. Specialist expertise and competence are expected in level 3, gained through high-level training and maintained through practice, continuous professional development and participation in professional networks (national or international) allowing exchange of ideas and experience [66]. In the USA, the United Council for Neurologic Subspecialities (UCNS) offers examination-based certification (and recertification) in headache medicine as a subspecialty [109], although there is no requirement for a physician practising as a “headache specialist” to be certified [110]. For some countries, training at this level will require attendance at specialist centres abroad. Several schemes exist for this. The University of Copenhagen and Danish Headache Centre, and Sapienza University in Rome, both offer Master degree courses in headache medicine, open to students from all countries [111, 112]. Training scholarships are offered by the International Headache Society (IHS) [113] and EHF (EHF-SAS [114]).

The major political and logistic implications of these requirements, especially at level 1 in view of the numbers of HCPs who need training, are probably the greatest barrier to nationwide implementation. Far-reaching national training must be part and parcel of effective headache-service reform, and it needs to commence in medical schools. Within the 3-level care system proposed, a training role for each higher level towards the level below can be envisaged (Fig. 2). The entire structure may depend upon these roles being developed.

### A comment on service quality

Effective service implementation does not of itself guarantee quality of care. Quality-assurance measures are necessary, as in all fields of medicine.

Standards and indicators exist for headache service quality evaluation (SQE), developed by LTB specifically to support implementation of structured headache services [108]. They are themselves undergoing evaluation at the various levels [66, 115–118].

### A comment on cost

Headache services delivering care equitably and nationwide to large numbers of people will undoubtedly enhance the headache patients’ journey and improve outcomes. They will also consume substantial health-care resources, and require major up-front investment. Cost-effectiveness becomes a key consideration. While preliminary economic analyses are highly favourable [21], there is a cost incurred by doing nothing.

There is promise of even greater savings to offset the cost – if improvements to services recover the lost productivity due to headache, or even a small part of it [11–19, 22]. This promise needs testing in formal economic analyses. If it proves to be sound, these savings foregone as a result of doing nothing become the financial penalty of inaction [29].

## Concluding remarks

Many problems beset the current compartmentalized division of headache services between primary, secondary and tertiary care. The model described seeks vertical integration. It recognizes that the demand for headache care dictates delivery for the most part in primary care, and that this is a perfectly good way forward in terms of accessibility and effectiveness of care. The model is amenable to horizontal integration with other care services, and capable of adaptation to suit local cultures and health-care systems.

The need for better – and better resourced – headache services exists in all countries, differing only quantitatively. At a time when momentum is again developing for health-service reform diverting resources from secondary to primary care [60, 77], there is opportunity for change. In low- and middle-income countries in particular, the growing shift of emphasis in health policy towards chronic non-communicable diseases [119] creates a fair climate for change.

Political will, needed for change to happen [9, 22, 60], will be driven by economic (cost-effectiveness) analyses. These, so far, have been highly encouraging [21], but more thorough evaluations are needed.

## Data Availability

Not applicable.

## Abbreviations

EHF: European Headache Federation
EU: European Union
FTE: Full-time equivalent
GBD: Global burden of disease (study)
GDP: Gross domestic product
GP: General practitioner
HCP: Health-care provider
ICHD: International classification of headache disorders
IHS: International Headache Society
LTB: *Lifting The Burden*
MOH: Medication-overuse headache
OTC: Over-the-counter
TTH: Tension-type headache
UdH: Undifferentiated headache
UK: United Kingdom
USA: United States of America
USSR: Union of Soviet Socialist Republics
WHO: World Health Organization
